# Cancer-associated fibroblast heterogeneity is associated with organ-specific metastasis in pancreatic ductal adenocarcinoma

**DOI:** 10.1186/s13045-021-01203-1

**Published:** 2021-11-02

**Authors:** Xingyi Pan, Jiaojiao Zhou, Qian Xiao, Kenji Fujiwara, Mengwen Zhang, Guanglan Mo, Wei Gong, Lei Zheng

**Affiliations:** 1 The Sidney Kimmel Comprehensive Cancer Center, Johns Hopkins University School of Medicine, Baltimore, MD USA; 2 Department of Oncology, Johns Hopkins University School of Medicine, 1650 Orleans Street, CRB1 Room 488, Baltimore, MD 21287 USA; 3 Department of Surgery, Johns Hopkins University School of Medicine, Baltimore, MD USA; 4 The Pancreatic Cancer Precision Medicine Center of Excellence Program, Johns Hopkins University School of Medicine, Baltimore, MD USA; 5 The Second Affiliated Hospital of the Zhejiang University, 88 Jie-Fang Rd, Hangzhou, 310009 China; 6 Present Address: Department of Surgery, Xinhua Hospital, Shanghai Jiaotong University, Shanghai, China

**Keywords:** DNA methylation, Cancer-associated fibroblast, Heterogeneity, Organ-specific metastasis, Pancreatic cancer

## Abstract

**Background:**

Metastasis occurs in the majority of pancreatic ductal adenocarcinoma (PDAC) patients at diagnosis or following resection. Patients with liver metastasis and those with lung metastasis have significantly different prognosis. Here, we sought to understand how cancer-associated fibroblasts (CAFs) play roles in the development of organ-specific metastasis.

**Methods:**

PDAC tumor cell lines established from the primary tumors with liver and lung metastasis potentials, respectively, in Kras/p53 mutation conditional knock-in (KPC) mice were co-cultured with matched CAFs or mouse mesenchymal stem cells. CAFs were isolated from metastases and subjected to DNA methylation and whole transcriptomic RNA sequencing analysis.

**Results:**

The ability of mouse PDAC tumor cell lines in developing liver or lung-specific metastases was demonstrated in orthotopic models. Tumor cells associated with liver metastasis potential, but not those associated with lung metastasis potential, induced the methylation of metabolism genes including NQO1 and ALDH1a3 and subsequent downregulated mRNA expression of a broader group of metabolism genes in CAFs. DNA methylation and downregulation of metabolism genes in CAFs in liver metastasis, but not those in lung metastasis, appeared to be regulated by DNA methyltransferase. Tumor cells associated with liver metastasis potential, but not those associated with lung metastasis potential, induce inflammatory CAF (iCAF) signatures. CAFs from liver metastasis demonstrated a more homogenous iCAF phenotype, whereas CAFs from lung metastasis maintained the heterogeneity.

**Conclusions:**

PDAC with organ-specific metastatic potentials has different capacities in inducing methylation of metabolism genes in CAFs, modulating CAF phenotypes, and resulting in different levels of heterogeneity of CAFs in different metastatic niches.

**Supplementary Information:**

The online version contains supplementary material available at 10.1186/s13045-021-01203-1.

To the editor

Our clinical observations revealed disease heterogeneity among pancreatic ductal adenocarcinoma (PDAC) patients with different distant metastatic sites, resulting in distinct clinical outcomes [1]. We sought to investigate the mechanism of organ-specific metastasis in the transgenic KPC mouse model [2] and established two cell lines from primary tumors of KPC mouse with liver metastasis only and KPC mouse with lung metastasis only (Fig. 1a; Additional file 1: Fig. S1A). Previously, we demonstrated that PDAC tumor cells induce DNA methylation globally in CAFs [3, 4] and in macrophages [5] including a wide range of metabolism genes resulting in their downregulation. We chose to examine methylation of *ALDH1*a3 in the glucose metabolism pathway and *NQO-1* in the oxidative phosphorylation (OXIPHOS) pathway (Additional file 1: Fig. S1B) and mRNA expression of their related genes (Additional file 1: Table S1). CAFs from liver metastases and lung metastases demonstrated different levels of methylation and mRNA expression in these genes (Fig. 1b–d).

**Fig. 1:**
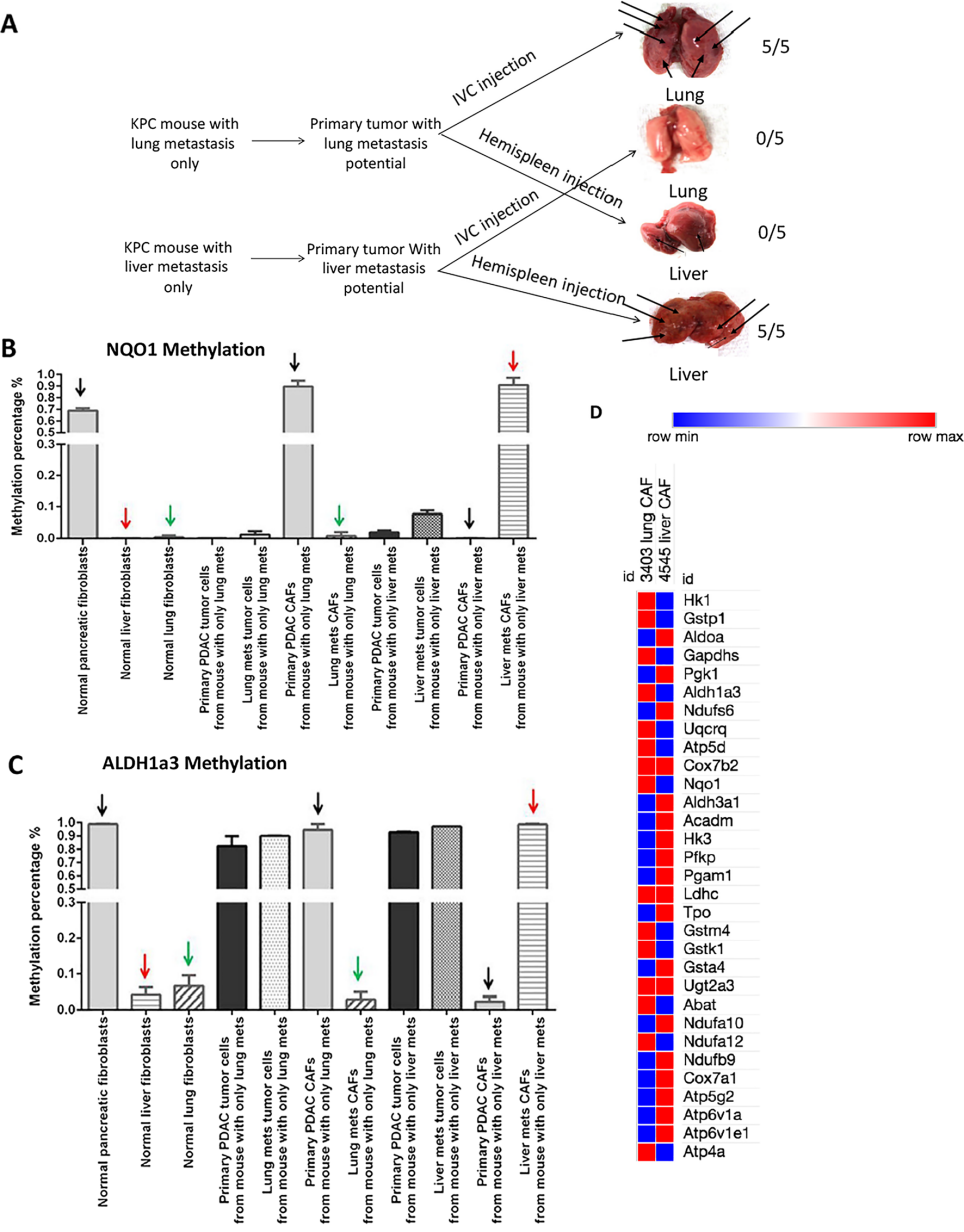
Distinct DNA methylation patterns in metabolism genes were observed in CAFs isolated from liver metastasis versus lung metastasis. **a** Establishment of primary mouse PDAC tumor cell lines with organ-specific metastasis potentials. Different KPC tumor cell lines were established from primary pancreatic tumor of KPC mice that developed liver metastasis only and lung metastasis only, respectively. These KPC cell lines were tested for their capacities to generate metastasis in mice through the hemispleen injection model of liver metastasis and the inferior vena cava injection model of lung metastases as described previously [6, 7]. Five mice per group were used to test each KPC cell line using both mouse models, respectively. Numbers of mice that developed large metastatic lesions were indicated. Longer arrows indicate large metastatic lesions; shorter arrows indicate small metastatic lesions. Note that the KPC cell line established from primary tumor with liver metastases consistently gave rise to liver metastasis macroscopically but not lung metastasis (5/5), whereas the KPC cell line established from primary tumor with lung metastases consistently gave rise to lung metastasis (5/5). The lung met tumor cell line was only able to develop a small number of macroscopically visualized metastatic foci when the cell line was injected by the hemispleen technique (Additional file 1: Fig. S1A). Liver and lung were examined microscopically and confirmed that the liver met tumor cell line only developed micro-metastases in lung (Additional file 1: Fig. S1A). **b** and **c** Percentages of methylation in the *ALDH1a3* and *NQO-1* gene were measured in tumor cells and CAFs isolated from primary pancreatic tumor and liver or lung metastases from KPC mouse with only liver or lung metastasis, respectively. Four different CAFs including: (1) CAFs isolated from primary KPC tumors of a mouse that developed liver metastasis only; (2) CAFs isolated from primary KPC tumors of a mouse that developed lung metastasis only; (3) CAFs isolated from liver metastases of a KPC mouse that developed liver metastasis only (liver mets CAFs); (4) CAFs isolated from lung metastases of a KPC mouse that developed lung metastasis only (lung mets CAFs) were all compared to normal pancreas (indicated with black arrow), liver (indicated with red arrow) and lung (indicated with green arrow) fibroblasts. Note that, for both *NQO-1* and *ALDH1a3* genes, DNA methylation levels were found to be elevated in liver mets CAFs from KPC mouse that developed liver metastasis compared to normal liver fibroblasts. *NQO-1* and *ALDH1a3* did not show an elevated DNA methylation in lung mets CAFs from KPC mouse that developed lung metastasis compared to normal lung fibroblasts, even though they demonstrated a high-level DNA methylation in CAFs from primary tumors that developed lung metastasis only. The methylation of *NQO-1* and *ALDH1a3* remained the same in tumor cells from both primary tumors and liver/lung metastases although the methylation level of *ALDH1a3* was higher than that of *NOQ-1* in tumor cells. Methylation percentage was quantified by MethySYBR real-time PCR (MSP). Black, red, green arrows indicate different comparison groups. Triplicate experimental results are presented as mean ± SEM. *Unpaired *t* test, *p* < 0.05. Independent experiments were conducted twice. **d** Heatmap was generated from the RNA sequencing analysis of mouse CAFs isolated from a KPC mouse that developed lung metastasis (3404LungCAF) only and from a KPC mouse that developed liver metastasis only (4545LiverCAF) to compare expression of selected metabolic genes. Mouse homologs of genes in the *ALDH1*-associated metabolism pathway and *NQO-1*-associated oxidative phosphorylation pathway previously found to have a significantly increased methylation level and also a significantly decreased mRNA expression level in CAFs following co-culture with human PDAC tumor cells [3] were selected. Heatmap was generated using transcripts per million (TPM) scores

We hypothesized that different methylation patterns in CAFs from different metastatic sites were induced by PDAC tumor cells with different metastasis potentials. We thus studied tumor-induced DNA methylation in the mouse mesenchymal stem cell line (moMSC), the precursor of CAFs, by co-culturing with three different primary PDAC tumor cell lines derived from a KPC mouse without metastasis (PancPrimaryTumorCell), a KPC mouse with liver metastasis only (LiverMetTumorCell), and a KPC mouse with lung metastasis only (LungMetTumorCell). Remarkably, different tumor cell lines showed differential capacities in inducing DNA methylation on *NQO-1* and *ALDH1a3* in moMSCs (Additional file 1: Fig. S2A and B). mRNA expression of *NQO-1* as well as other metabolism genes were also downregulated in moMSC co-cultured with LiverMetTumorCells (Additional file 1: Fig. S2C–E). These results suggested that different PDAC tumor cells show different capacities in possibly shaping metabolic states of CAFs in different metastatic sites. We also developed CAF lines from primary KPC tumor with liver metastasis only (4545PancCAF) or that with lung metastasis only (3403PancCAF) and co-cultured them with matched tumor cells. Induced DNA methylation changes in *NQO1* and *ALDH1a3* were observed at higher degrees in 4545PancCAF from tumor co-culture compared to its mono-culture than those in 3403PancCAF (Additional file 1: Fig. S2F). Consistently, *NQO-1* expression was downregulated in 4545PancCAF from tumor co-culture (Additional file 1: Fig. S2G). *ALDH1a3* expression was not downregulated in 4545PancCAF from tumor co-culture, suggesting that *ALDH1a3* expression is regulated by a more complex mechanism (Additional file 1: Fig. S2H).

We next sought to understand if tumor-induced gene methylation occurred in the metastatic niches where tumor cells metastasized to. We orthotopically implanted KPC cells with both liver and lung metastasis potentials (Fig. 2a). The transplanted mice developed liver and/or lung metastases. *NQO-1* methylation was elevated in CAFs isolated from liver metastases compared to normal liver fibroblasts (Fig. 2b), but remained at baseline levels in CAFs from lung metastases (Fig. 2c). Supporting this notion, tumor-induced methylation of *NQO-1* and *ALDH1a3* and downregulation of their mRNA expression or expression of their related metabolism genes in CAFs from liver metastasis could be reversed by treatment of DNA demethylating agent, decitabine, whereas methylation in CAFs from lung metastasis was not affected (Additional file 1: Fig. S3). Thus, although the primary tumors harbor an organ-specific metastatic potential, metastatic organs possibly harbor contributive factors for an organ-specific tumor-induced metabolism gene methylation.

**Fig. 2:**
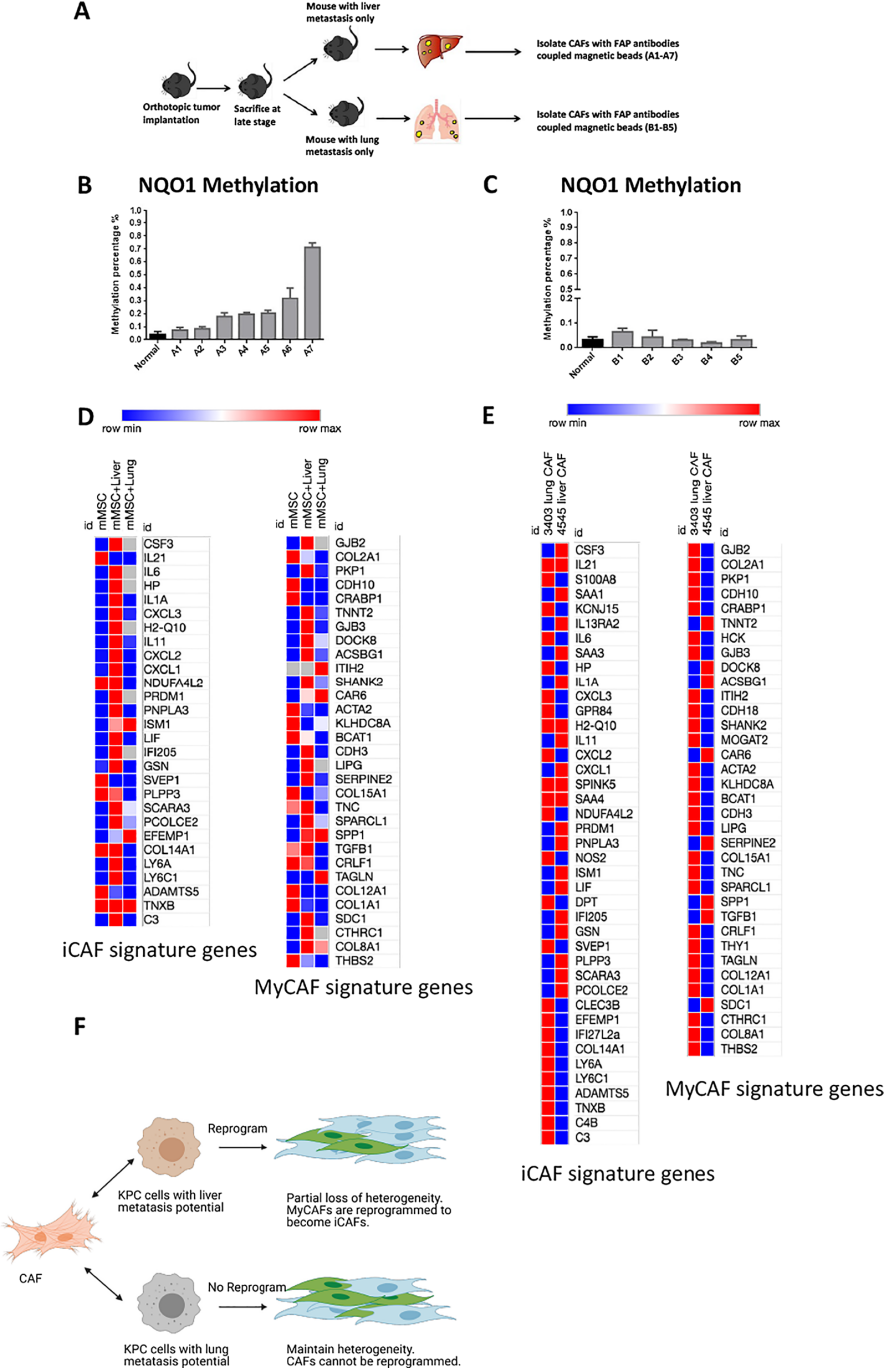
PDAC tumor cells with different organ-specific metastatic potentials showed different capacities in modulating the metabolism gene methylation in CAFs and also the heterogeneity of CAFs in metastatic sites. **a** The schema shows that wild-type C56Bl/6 mice orthotopically implanted with primary pancreatic KPC tumors spontaneously developed liver or lung metastases, which were dissected for CAF isolation by using magnetic beads coupled with anti-FAP antibodies. Two KPC cell lines from mice that developed both liver and lung metastases were orthotopically implanted in the pancreas of 40 mice (20 mice per cell line). As previously shown [6, 7], the majority of mice died from primary tumor growth without metastases. Of 40 mice, 7 of them developed liver metastasis only and 5 developed lung metastasis only. CAFs were isolated from dissected liver and lung metastases. **b** Percentage of methylation in the *NQO-1* gene in CAFs from multiple liver metastases was measured by MSP. Methylation percentage of *NQO-1* in normal liver fibroblasts was used as a baseline methylation level for comparison. A1-7: 7 mice developed liver metastasis spontaneously. Note that methylation levels of the *NQO-1* gene were elevated in CAFs isolated from liver metastases compared to normal liver fibroblasts. **c** Percentage of methylation in the *NQO1* gene in CAFs from multiple lung metastases was measured by MSP. Methylation percentage of *NQO-1* in normal lung fibroblasts was used as a baseline methylation level for comparison. B1-5: 5 mice developed lung metastasis spontaneously. Note that the gene methylation levels of *NQO-1* remained at baseline levels in CAFs isolated from lung metastases compared to normal lung fibroblasts. **d** Heatmap was generated using TPM scores based on the RNA sequencing analysis of mouse moMSC (mMSC) after co-cultured with Liver Met tumor cells (mMSC + liver) and Lung Met tumor cells (mMSC + lung) to compare the expression of iCAF and myCAF signature genes. Red color indicates upregulation in co-culture compared to mono-culture, blue color indicates downregulation in co-culture compared to mono-culture. **e** Heatmap was generated using TPM based on the RNA sequencing analysis of mouse CAFs isolated from liver metastasis (4545 liver CAF) and from lung metastasis only (3403 lung CAF) to compare the expression of iCAF and myCAF signature genes. **f** Diagram illustration shows that CAFs in liver metastasis are reprogrammed by PDAC cells with liver metastasis potential and subsequently lose part of their heterogeneity. Note that CAFs in lung metastasis are not reprogrammed by KPC cells with lung metastasis potential and thus maintain their heterogeneity. Image created using Biorender

Nevertheless, it was unknown whether CAFs from different metastatic niches are functionally different. The functions of the subpopulations of CAFs were previously characterized and showed different transcriptomic signatures [8–10]. In our RNA sequencing analysis, mono-cultured moMSCs showed low expression of the majority of inflammatory CAF (iCAF) signature genes (Additional file 1: Table S2) and high expression of some of myofibroblastic CAF (myCAF) signature genes (Fig. 2d). However, upon co-cultured with LiverMetTumorCells, moMSC was programmed to express essentially all iCAF signature genes and the majority of myCAF signature genes. However, expression of the majority of iCAF and myCAF genes in moMSC co-cultured with LungMetTumorCell remained low, suggesting that LungMetTumorCell was not able to program moMSC. As expected, CAFs from lung metastasis expresses both iCAF genes and myCAF genes (Fig. 2e). By contrast, CAFs from liver metastasis did not demonstrate a myCAF signature, but expressed some iCAF genes that were not upregulated in CAFs from lung metastasis. Taken together, CAFs in liver metastasis are more homogeneous likely as a result of reprogramming of myCAF into iCAF by tumor cells that metastasize to liver, whereas CAFs in lung metastasis remain to be heterogenous (Fig. 2f). A change in the heterogeneity of CAFs to a more homogeneous iCAF phenotype may be responsible for the aggressive feature of liver metastasis and is potentially targetable by anti-IL-1β antibody [11].

## Supplementary Information

**Additional file 1**. Representative images of lung and liver metastases from KPC mice implanted with KPC tumor cells with lung and liver metatasis potentials and schema of how represntative metabolism genes were selected for study.

## Data Availability

RNA sequencing data are provided as supplement materials.

## Abbreviations

PDAC: Pancreatic ductal adenocarcinoma
CAFs: Cancer-associated fibroblasts
KPC: Kras/p53 mutation conditional knock-in
moMSC: Mouse mesenchymal stem cells
RNAseq: RNA sequencing
iCAF: Inflammatory CAF
myCAFs: Myofibroblastic CAFs
apCAFs: Antigen-presenting CAFs
TME: Tumor microenvironment
