# Supplementary material for: Cancer-associated fibroblast heterogeneity is associated with organ-specific metastasis in pancreatic ductal adenocarcinoma
Source: J Hematol Oncol. 2021 Nov 2;14:184. doi: 10.1186/s13045-021-01203-1 (PMC8561960; doi:10.1186/s13045-021-01203-1)
Supplement: Supplementary file 1 — Additional file 1. Representative images of lung and liver metastases from KPC mice implanted with KPC tumor cells with lung and liver metatasis potentials and schema of how represntative metabolism genes were selected for study. [file 13045_2021_1203_MOESM1_ESM.docx]

**Additional file 1**

**Cancer-associated fibroblast heterogeneity is associated with organ-specific metastasis in pancreatic ductal adenocarcinoma**

**Xingyi Pan^1,2,4^**^†^**, Jiaojiao Zhou^1,2*^**^†^**, Qian Xiao^1,2^, Kenji Fujiwara^1,2,4^, Mengwen Zhang^1,2,4^, Guanglan Mo^1,2,4^, Wei Gong^1,2,3#^, Lei Zheng ^1,2,3,4*^**

**Affiliations:**

^1^ The Sidney Kimmel Comprehensive Cancer Center, Johns Hopkins University School of Medicine, Baltimore Maryland, USA

^2^ Department of Oncology, Johns Hopkins University School of Medicine, Baltimore Maryland, USA

^3^ Department of Surgery, Johns Hopkins University School of Medicine, Baltimore Maryland, USA

^4^The Pancreatic Cancer Precision Medicine Center of Excellence Program, Johns Hopkins University School of Medicine, Baltimore, Maryland, USA

**Running Title:**

CAF heterogeneity in organ-specific metastasis in PDA

***Corresponding Authors:**

Lei Zheng, MD., Ph.D.,

1650 Orleans Street, CRB1 Room 488,

Baltimore, MD 21287

Tel: 410-502-6241

Fax: 410-614-8216

Email: [lzheng6@jhmi.edu](mailto:lzheng6@jhmi.edu)

Jiaojiao Zhou, M.D.

The Second Affiliated Hospital of the Zhejiang University

88 Jie-Fang Rd, Hangzhou, China, 310009

Tel: +86-571-87784527

Fax: +86-571-87784501

Email: [zhoujj@zju.edu.cn](mailto:zhoujj@zju.edu.cn)

**†** These authors contribute equally to this work and should be considered as first authors.

^#^Present Address: Department of Surgery, Xinhua Hospital, Shanghai Jiaotong University, Shanghai, China

**Additional file 1: Methods:**

**Cell lines and culture conditions**

**KPC cell line**

The genetically engineered KPC mouse model with the Kras and p53 mutation conditional knock-in allele under the control of PDX1-cre was previously backcrossed to the C57Bl/6 background(1,2). A KPC tumor cell line was previously derived from a KPC mouse that did not demonstrate metastasis. In this study, four additional KPC tumor cell lines were established from primary tumors of four KPC mice as described previously(3), one from a KPC mouse with only liver metastases (mouse#4545) identified macroscopically and microscopically, a second from a KPC mouse with only lung metastases (mouse#3403) identified macroscopically and microscopically, and the other two from KPC mice with both liver and lung metastases identified macroscopically and microscopically. KPC cell line established from mouse 4545 with liver metastases was named as 4545 Liver Met tumor cell. KPC cell line established from mouse 3403 with lung metastases was named as 3403 Lung Met tumor cell. The PDAC cell lines were cultured in RPMI 1640 media containing 10% FBS, 1 mM sodium pyruvate, 2mM L-glutamine, 1% nonessential amino acids (100×) and 50 units/mL penicillin, 50 µg/mL streptomycin (Life Technology, Carlsbad, CA, USA).

**Mouse mesenchymal stem cell (MSC) cell line**

Mouse MSC cells were obtained from Texas A&M Health Science Center (<http://medicine.tamhsc.edu/irm/msc-distribution.html>). MSC cells were cultured in Alpha Modified Eagle Medium media containing 10% premium selected Fetal Bovine Serum (FBS)(Atlanta Biologicals) and passaged for fewer than 6 months after resuscitation in 2013 and authenticated by DNA and gene expression profiling.

**Primary CAF line establishment**

Primary CAF lines (3403 Lung CAF and 4545 liver CAF) were established from surgically resected lung and liver metastases of KPC mice (#3043 and #4545) and authenticated by DNA and gene expression profiling as previously described(4). In addition, primary tumor and CAF cultures were obtained from matched primary PDAC tumors and liver metastases or lung metastase of multiple KPC mice without developing them into cell lines by following the same protocols.

**Hemispleen model of liver metastasis**

The mouse hemi-spleen liver metastasis model has been previously described(5,6). In short, the spleens of anesthetized female C57Bl/6 mice of ages 8 to 10 weeks were divided into two halves, and the halves were clipped at the cutting edge. 2 × 10^5^ of KPC cells with liver and lung potential were injected into one hemi-spleen followed by a flush of phosphate-buffered saline (PBS) buffer. People who performed surgery were blinded about what types of KPC cells were used during the injection. After the injection, the hemi-spleen was removed. The abdominal wall was sutured, and the skin was adapted using wound clips. All mice were randomized after surgery completion for further experiments and were followed twice daily for survival. The mice were sacrificed 19 days after the hemispleen injection, and the livers were harvested for histological analysis of metastasis formation. Five mice per group were included in each experiment which was repeated at least twice.

**Inferior vena cava injection model of lung metastases**

The inferior vena cava (IVC) injection model of lung metastases has been previously described(7). In short, 5 × 10^5^ KPC cells with liver or lung metastasis potential were injected into the IVC toward pulmonary veins at a position above the superior mesenteric vein of anesthetized female C57Bl/6 mice of ages 8 to 10 weeks. People who performed surgery were blinded from what types of KPC cells were used during the injection. The abdominal wall was then sutured, and the skin was adapted using wound clips. All mice were randomized after surgical implantation for further experiments and were followed twice daily for survival. The mice were sacrificed 19 days after the IVC injection, and the lungs were harvested for histological analysis of metastasis formation. Five mice per group were included in each experiment which was repeated at least twice.

At necropsy, metastases were examined. Both macro-metastases and micro-metastases were scored for all metastatic evaluations. All macro-metastases observed during necropsy were confirmed upon histological analysis. Additional micro-metastases were found through histological examination of the tissue sections.

**Orthotopic tumor implantation model**

The mouse pancreatic orthotopic model was described previously(8). In brief, 2 × 10^6^ PDA cells were subcutaneously injected into two flanks of syngeneic female C57Bl/6 mice. Subcutaneous tumors were harvested and cut into 2-mm^3^ pieces after 2 weeks for tumor implantation on the pancreas of syngeneic female C57Bl/6 mice at ages between 8 to 10 weeks. All mice were followed twice daily for survival. Tumor metastasis was visualized and dissected for CAF isolation. Ten mice per group were included in each experiment which was repeated at least twice. Survival was the endpoint of the experiment or mice were euthanized according to the animal protocol when they developed morbidity.

**Fibroblasts processing and isolation**

CAFs and normal fibroblasts isolation were described previously(9). In brief, liver or lung metastases were visualized and dissected into collection tubes from spontaneous tumor developing KPC mice or tumor inoculation by the orthotopic tumor implantation. Normal liver and lung were also collected. They were mechanically processed, suspended in ACK lysis buffer (Quality Biological) and subsequently resuspended in 80% Percoll (GE Healthcare LifeSciences) and 40% Percoll. After preparing the cells into single cell suspension, CAFs or fibroblasts were isolated using direct technique isolation using Cellection Biotin Binder kit (Life Technologies) and the sheep anti-human FAP biotinylated affinity purified antibody (R&D Systems Inc. Minneapolis, MN, USA) according to the Direct Technique isolation instructions.

**In vitro co-culture experiment**

MoMSCs or CAFs and PDAC tumor cells were plated at cell density ratio of 1:3, with 1×10^6^ cells moMSCs or CAF cells and 3×10^6^ PDAC tumor cells into a T75 culture flask for 24 hours. After co-culturing, moMSCs or CAFs isolation was performed using the Cellection Biotin Binder Kit (Life Technology, Carlsbad, CA, USA) and the sheep anti-human FAP biotinylated affinity purified antibody (R&D Systems Inc. Minneapolis, MN, USA) according to the Direct Technique isolation instructions.

### 5-Aza-2’-deoxycytidine(Decitabine; DAC) treatment

Cells were plated at cell denticity of 1×10^6^ cells per T75 flask and treated with 1 µmol/L Decitabine, DAC (Sigma Aldrich) consecutively for 72 hours, with changes of media and drug every 24 hours. Cells were harvested at the end of the 3-day treatment course for co-culture experiment.

### Bisulfite conversion and methylation specific PCR

Genomic DNA extraction from Dynabeads bound cells was performed using QIAamp DNA micro kit(Qiagen). DNA from tumor or CAF cultures was extracted using Blood and Tissue DNeasy kit(Qiagen). Extracted DNA was bisulfite-modified using EZ DNA methylation Kit(Zymo Research) which converted all unmethylated cytosines to uracils while leaving methyl-cytosines intact. After bisulfite conversion single stranded DNA was amplified using methylation specific PCR(MSP) as previous described(10) using StepOnePlus Real-Time PCR System (Thermo Scientific, Hudson, NH, USA). The primers and PCR conditions for MSP are shown in **Additional file 1: Table S4**. The MSP methylation percentage was calculated as: [1/(1+2^(CT Meth value–CT Umeth value) ] ×100%. All MSP reactions were conducted as triplicate. Independent experiments were repeated twice.

**RNA preparation and quantitative real-time PCR**

Total RNA was extracted with the TRIzol reagent (Life Technology, Carlsbad, CA, USA) according to manufacturer’s guide. cDNA was synthesized by ReadyScript® cDNA Synthesis kit (Sigma, St. Louis, MO, USA). Quantitative real-time RT-PCR (qRT-PCR) analysis was performed on the StepOnePlus Real-Time PCR System (Thermo Scientific, Hudson, NH, USA). All the primers we used were listed in the **Additional file 1: Table S3**. The expression of genes was quantified by SYBR Green (Applied Biosystems) fluorescence. All gene expression was normalized to the expression of β-actin. All PCR reactions were performed in triplicate. Independent experiment were repeated twice.

**RNA sequencing analysis**

Total RNAs were isolated from CAFs and moMSCs using the TRIzol reagent (Life Technology, Carlsbad, CA, USA) according to manufacturer’s guide. RNA-seq library was prepared by the BGI Genomics Commercial Service. Sequence reads were obtained using BGIseq500 (Illumina) and successfully mapped to mouse genome. Reads counts were normalized based on RPKM, fold changes were calculated for all possible comparisons and a 1.5-fold cutoff was used to select genes with expression changes. Heatmap was generated using Morpheus(<https://software.broadinstitute.org/morpheus/>).

**Quantification and statistical analysis**

Data are presented as means ± SEM. Student T-test and Chi-square test were used for categorical variable comparison between two unpaired groups. All tests were two-tailed, and p < 0.05 was considered statistically significant. Statistical analyses were done using GraphPad Prism 7.0 software (GraphPad Software).

**Study Approval**

All studies and maintenance of mice were conducted in accordance with the approval of the Institutional Animal Care and Use Committee (IACUC) guidelines at Johns Hopkins.

**
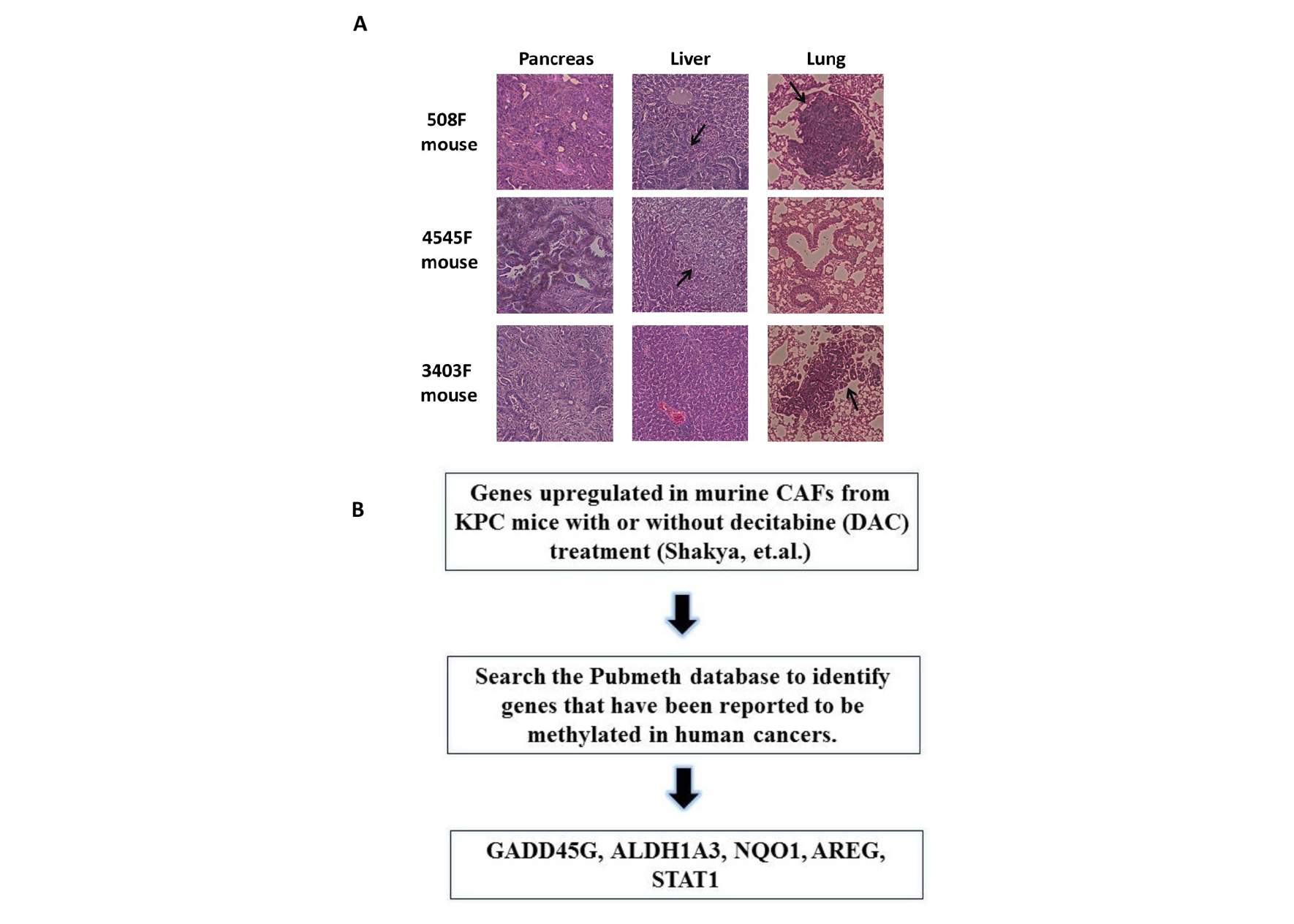
Additional file 1: Figures and Tables**

**Additional file 1: Figure S1. (A)** Representative H&E staining of pancreas, liver and lung of KPC mouse that spontaneously developed liver metastasis (4545F mouse), lung metastasis (3403F mouse) and mouse that developed both liver and lung metastasis (508F mouse). 4545F mouse was later used to develop KPC tumor cell line with liver metastasis potential while 3403F mouse was used to develop KPC tumor cell line with lung metastasis potential. **(B)** The schema shows how the candidate metabolic genes for methylation regulation were selected.


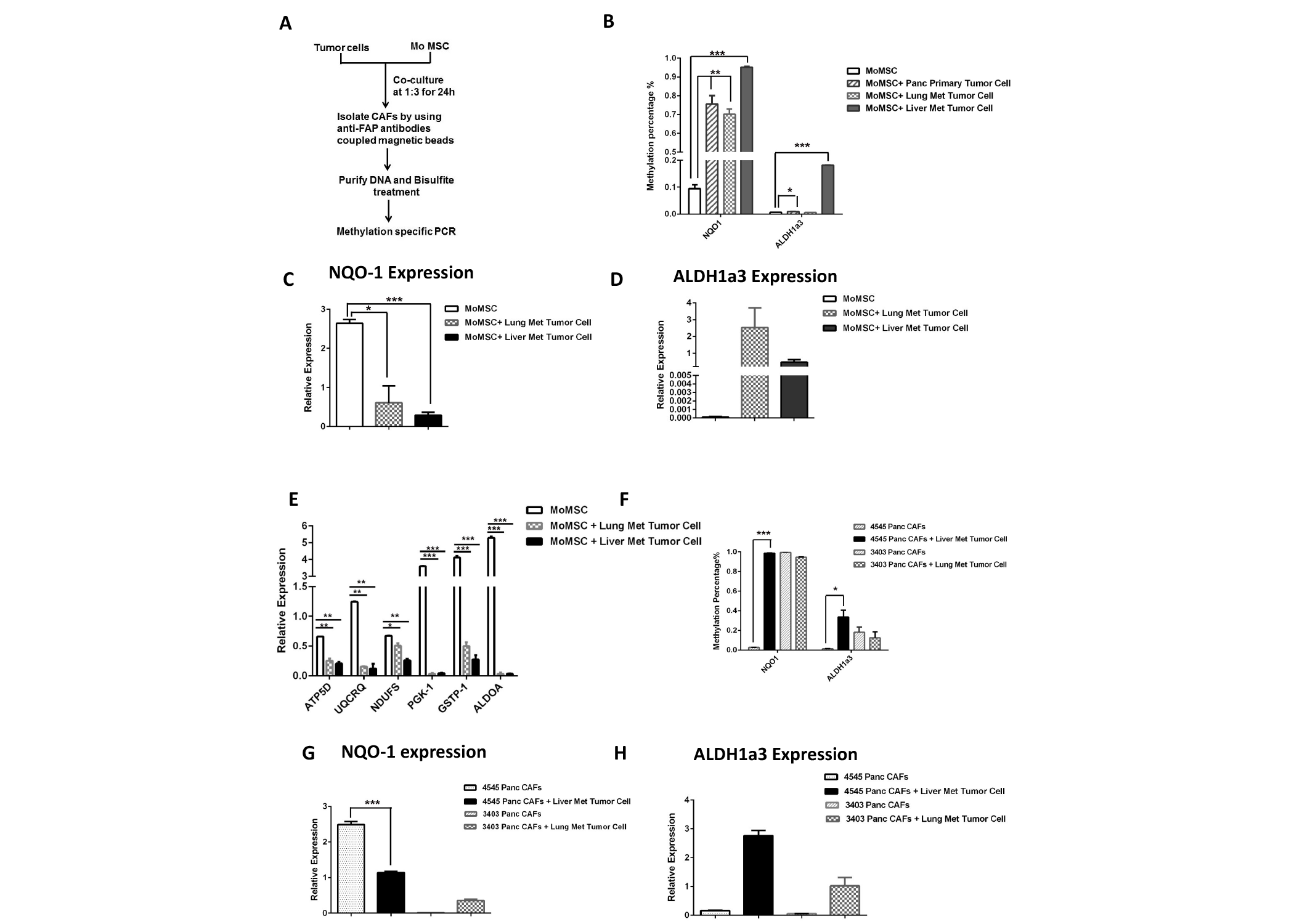


**Additional file 1: Figure S2. PDAC tumor cells associated with liver metastasis potential reprogrammed the metabolism gene methylation and expression in CAFs differently from those associated with lung metastasis potential. (A)** Experimental schema shows that mouse mesenchymal stem cells (moMSC) were co-cultured with KPC tumor cells in a ratio of 1:3 for 24 hours, followed by isolation of moMSC using magnetic beads coupled with anti-FAP antibodies. **(B)** Percentages of methylation in the ALDH1a3 and NQO-1genes were measured in moMSC cells in mono-culture and in co-culture with PDAC tumor cells with liver metastasis potential (Liver Met tumor cell)and PDAC tumor cells with lung metastasis potential (Lung Met tumor cell), respectively. ALDH1a3 and NQO-1 methylation were quantified using MSP. Triplicate experiment results are presented as mean ± SEM. Unpaired t-test, *: p<0.05, ** : p<0.01,*** : p<0.001. **(C)(D)** NQO-1, ALDH1a3 expression were measured using real-time(RT)-PCR in mouse moMSC cells in mono-culture and in co-culture with Liver Met tumor cells and Lung Met tumor cells, respectively. Expression fold change was normalized and calculated by using β-actin as internal control and moMSC cells in mono-culture as sample comparison control. Triplicate experiment results are presented as mean ± SEM. Unpaired t-test, *: p<0.05, ** : p<0.01,*** : p<0.001. Independent experiments were conducted twice. **(E)** Expression of key metabolic genes including UQCRQ, ATP5D, NDUFS6, GSTP-1 were measured for comparison in moMSC cells in mono-culture and in co-culture with Liver Met tumor cells and Lung Met tumor cells, respectively, using RT-PCR. **(F)** Methylation percentages of ALDH1a3 and NQO-1 were measured in 4545 Panc CAFs isolated from primary pancreatic tumor of 4545 KPC mouse(liver metastasis only) in mono-culture and in co-culture with its matching liver Met tumor cells, 3403 Panc CAFs isolated from primary pancreatic tumor of 3403 KPC mouse(lung metastasis only) in mono-culture and in co-culture with its matching lung Met tumor cells. **(G)(H)** mRNA expression of NQO-1 and ALDH1a3 were measured in 4545 Panc CAFs in mono-culture and in co-culture with its matching liver Met tumor cells, 3403 Panc CAFs in mono-culture and in co-culture with its matching lung Met tumor cells. RNA expression fold change was normalized and calculated by using β-actin as internal control and 4545, 3403 CAFs in mono-culture as sample comparison control, respectively.


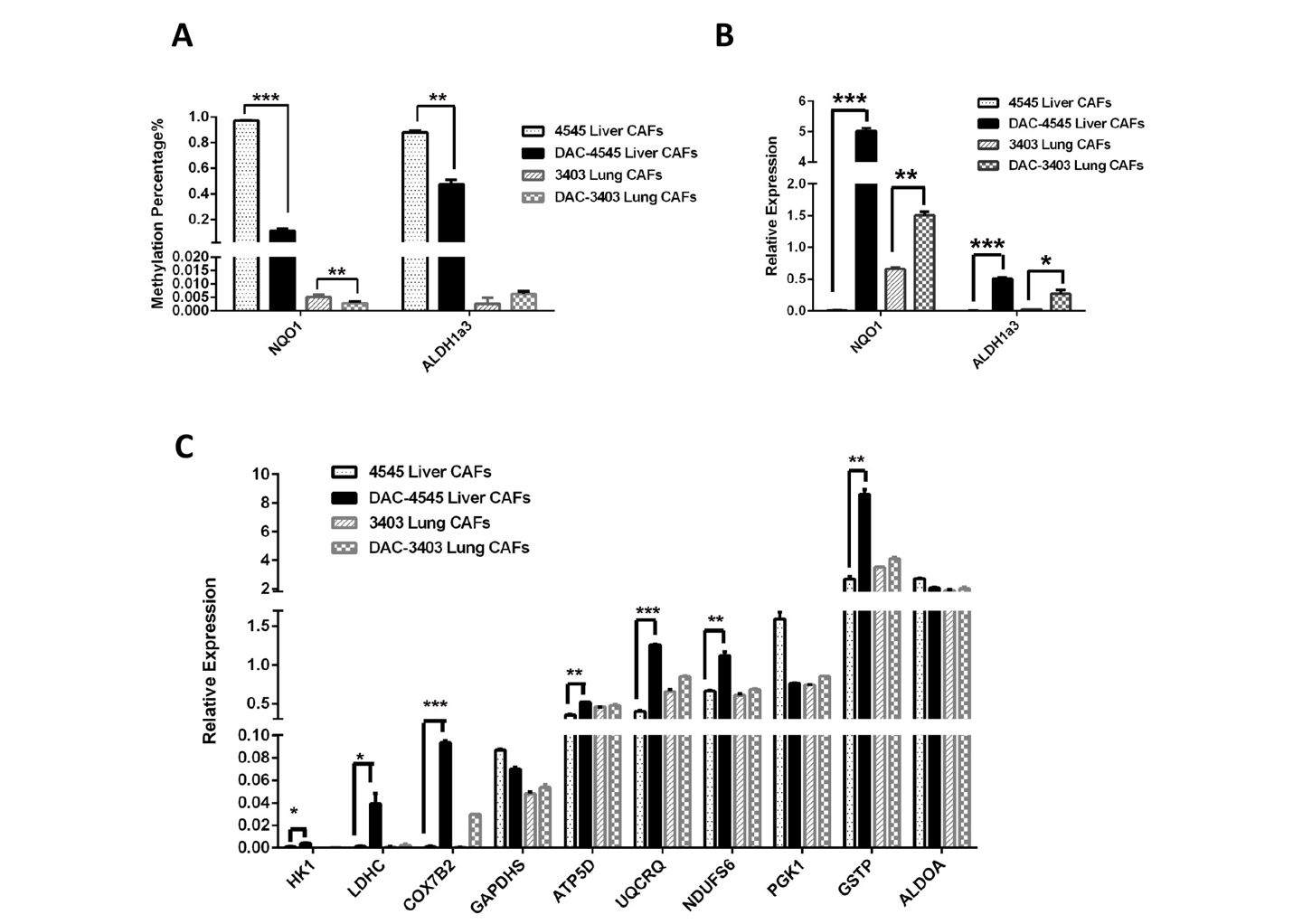


**Additional file 1: Figure S3.** **DNA methylation and downregulation of metabolism genes in CAFs in liver metastasis, but not those in lung metastasis, were regulated by the DNA methyltransferase inhibitor.** CAFs from liver metastases of the KPC mouse that develop liver metastasis(4545 Liver CAF) and CAFs from lung metastases of the KPC mouse that develop lung metastasis(3403 Lung CAF) were treated with or without Decitabine at concentration of 1 µmol/L for 72 hours before being co-cultured with matched primary KPC PDAC tumor cells for 24 hours or mono-cultured as a technical control for 24 hours and were subsequently re-isolated from co-culture. Note that CAFs were pre-treated with Decitabine, which would not exert an effect on tumor cells in the subsequent co-culture. (**A)** Methylation level of NQO1 and ALDH1a3 in 4545 Liver CAFs and 3403 Lung CAFs with and without treatment of Decitabine(DAC) and subsequently co-cultured with matched primary KPC PDAC cells was measured. Note that DNA methylation of NQO-1 and ALDH1a3 in 4545 Liver CAFs from liver metastasis was significantly reduced after DAC treatment compared to untreated CAFs while no statistically significant DNA methylation changes were observed in treated or untreated 3403 Lung CAFs from lung metastasis. **(B)** mRNA expression of NQO1 and ALDH1a3 in 4545 Liver CAFs and 3403 Lung CAFs with and without DAC treatment and subsequently co-cultured with matched primary KPC PDAC cells was measured. Expression fold change was normalized and calculated by using β-actin as internal control and 4545, 3403 CAFs without DAC treatment as sample comparison control, respectively. Note that gene expression of NQO-1 and ALDH1a3 at mRNA level was upregulated at higher levels in 4545 Liver CAFs associated with liver metastasis after DAC treatment than that in 3403 Lung CAFs. **(C)** mRNA expression of HK-1, LDHC, COX7B2, ATP5D, UQCRQ, NDUFS6 and GSTP1 in 4545 Liver CAFs and 3403 Lung CAFs with and without DAC treatment and subsequently co-cultured with matched primary KPC PDAC cells was measured. Expression fold change was normalized and calculated by using β-actin as internal control and 4545, 3403 CAFs without DAC treatment as sample comparison control, respectively. Note that gene expression of metabolic genes including HK-1, LDHC, COX7B2, ATP5D, UQCRQ, NDUFS6 and GSTP1 was significantly upregulated in 4545 Liver CAFs from liver metastasis compared to 3403 Lung CAFs from lung metastasis after DAC treatment. For **A-C**, triplicate experiment results are presented as mean ± SEM. Unpaired t-test, *: p<0.05, ** : p<0.01,*** : p<0.001.

**Additional file 1: Table S1. Selection of metabolism genes**

**Additional file 1: Table S2. Selected top 42 most highly expressed iCAF signature genes and top 40 most highly expressed myCAF signature genes.**

**Additional file 1: Table S3.** **RT-PCR primers.**

**
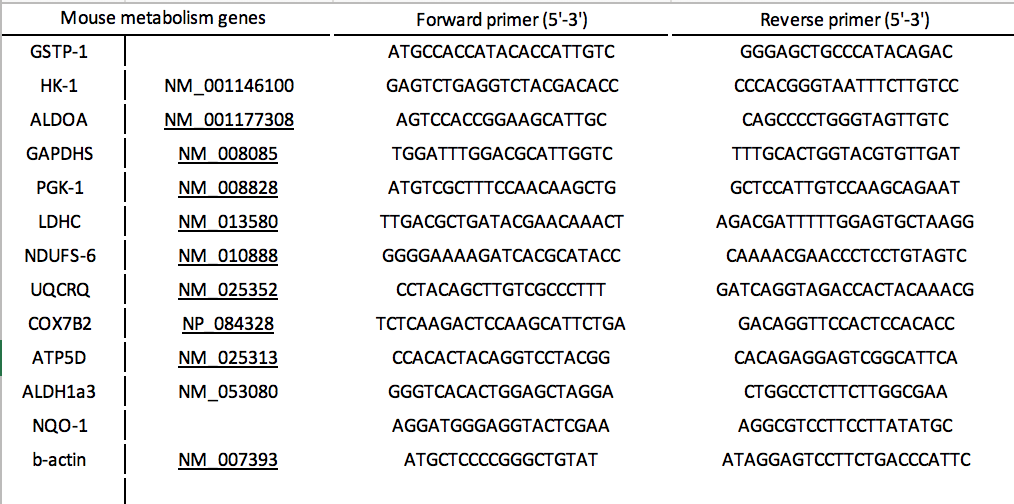
**

**Additional file 1: Table S4.** **MSP primers**


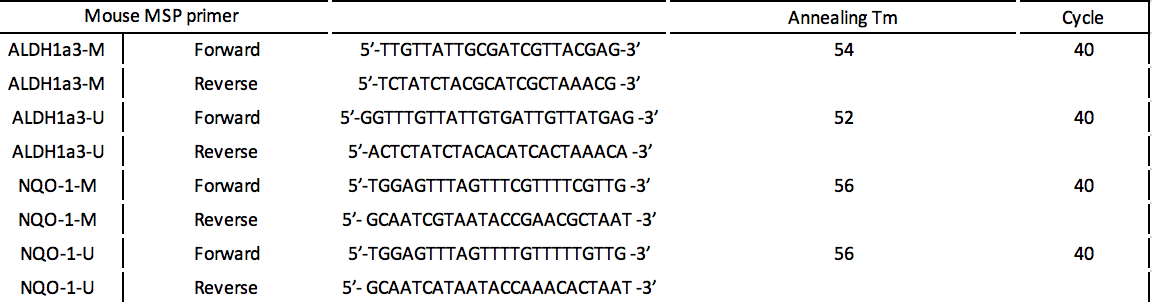


**References in Additional file 1**

1. Hingorani SR, Wang L, Multani AS, Combs C, Deramaudt TB, Hruban RH*, et al.* Trp53R172H and KrasG12D cooperate to promote chromosomal instability and widely metastatic pancreatic ductal adenocarcinoma in mice. Cancer Cell **2005**;7(5):469-83 doi 10.1016/j.ccr.2005.04.023.

2. Lee JW, Komar CA, Bengsch F, Graham K, Beatty GL. Genetically Engineered Mouse Models of Pancreatic Cancer: The KPC Model (LSL-Kras(G12D/+) ;LSL-Trp53(R172H/+) ;Pdx-1-Cre), Its Variants, and Their Application in Immuno-oncology Drug Discovery. Current protocols in pharmacology **2016**;73:14.39.1-14.39.20 doi 10.1002/cpph.2.

3. Orth M, Metzger P, Gerum S, Mayerle J, Schneider G, Belka C*, et al.* Pancreatic ductal adenocarcinoma: biological hallmarks, current status, and future perspectives of combined modality treatment approaches. Radiation Oncology **2019**;14(1):141 doi 10.1186/s13014-019-1345-6.

4. Seppänen H, Juuti A, Mustonen H, Haapamäki C, Nordling S, Carpelan-Holmström M*, et al.* The Results of Pancreatic Resections and Long-Term Survival for Pancreatic Ductal Adenocarcinoma: A Single-Institution Experience. Scandinavian Journal of Surgery **2016**;106(1):54-61 doi 10.1177/1457496916645963.

5. Soares KC, Foley K, Olino K, Leubner A, Mayo SC, Jain A*, et al.* A preclinical murine model of hepatic metastases. J Vis Exp **2014**(91):51677 doi 10.3791/51677.

6. Jain A, Slansky JE, Matey LC, Allen HE, Pardoll DM, Schulick RD. Synergistic effect of a granulocyte-macrophage colony-stimulating factor-transduced tumor vaccine and systemic interleukin-2 in the treatment of murine colorectal cancer hepatic metastases. Ann Surg Oncol **2003**;10(7):810-20 doi 10.1245/aso.2003.10.006.

7. Foley K, Rucki AA, Xiao Q, Zhou D, Leubner A, Mo G*, et al.* Semaphorin 3D autocrine signaling mediates the metastatic role of annexin A2 in pancreatic cancer. Sci Signal **2015**;8(388):ra77 doi 10.1126/scisignal.aaa5823.

8. Zheng L, Foley K, Huang L, Leubner A, Mo G, Olino K*, et al.* Tyrosine 23 phosphorylation-dependent cell-surface localization of annexin A2 is required for invasion and metastases of pancreatic cancer. PLoS One **2011**;6(4):e19390 doi 10.1371/journal.pone.0019390.

9. Blair AB, Kim VM, Muth ST, Saung MT, Lokker N, Blouw B*, et al.* Dissecting the Stromal Signaling and Regulation of Myeloid Cells and Memory Effector T Cells in Pancreatic Cancer. Clinical Cancer Research **2019**;25(17):5351 doi 10.1158/1078-0432.CCR-18-4192.

10. House MG, Guo M, Iacobuzio-Donahue C, Herman JG. Molecular progression of promoter methylation in intraductal papillary mucinous neoplasms (IPMN) of the pancreas. Carcinogenesis **2003**;24(2):193-8 doi 10.1093/carcin/24.2.193.
